# Reduction and recovery of self-sustained muscle activity after fatiguing plantar flexor contractions

**DOI:** 10.1007/s00421-023-05403-0

**Published:** 2024-02-10

**Authors:** Anthony J. Blazevich, Ricardo N. O. Mesquita, Ronei S. Pinto, Timothy Pulverenti, Sébastien Ratel

**Affiliations:** 1 School of Medical and Health Sciences, Centre for Human Performance, Edith Cowan University, Joondalup, Australia; 2 Department of Electrical Engineering, Chalmers University of Technology, Gothenburg, Sweden; 3 Neuroscience Research Australia, Sydney, Australia; 4 Exercise Research Laboratory, School of Physical Education, Physiotherapy and Dance, Universidade Federal do Rio Grande do Sul, Porto Alegre, Brazil; 5 Department of Physical Therapy, College of Staten Island, Staten Island, NY USA; 6 UFR STAPS - Laboratoire AME2P, Université Clermont Auvergne, Campus Universitaire des Cézeaux, 3 Rue de la Chebarde, 63170 Clermont-Ferrand, France

**Keywords:** Fatigue, Muscle strength, Maximum voluntary contraction, Neuromuscular, PIC

## Abstract

**Purpose:**

Persistent inward calcium and sodium currents (PICs) are crucial for initiation and maintenance of motoneuron firing, and thus muscular force. However, there is a lack of data describing the effects of fatiguing exercise on PIC activity in humans. We simultaneously applied tendon vibration and neuromuscular electrical stimulation (VibStim) before and after fatiguing exercise. VibStim induces self-sustained muscle activity that is proposed to result from PIC activation.

**Methods:**

Twelve men performed 5-s maximal isometric plantar flexor contractions (MVC) with 5-s rests until joint torque was reduced to 70%MVC. VibStim trials consisted of five 2-s trains of neuromuscular electrical stimulation (20 Hz, evoking 10% MVC) of triceps surae with simultaneous Achilles tendon vibration (115 Hz) without voluntary muscle activation. VibStim was applied before (PRE), immediately (POST), 5-min (POST-5), and 10-min (POST-10) after exercise completion.

**Results:**

Sustained torque (T_sust_) and soleus electromyogram amplitudes (EMG) measured 3 s after VibStim were reduced (T_sust_: −59.0%, *p* < 0.001; soleus EMG: −38.4%, *p* < 0.001) but largely recovered by POST-5, and changes in MVC and T_sust_ were correlated across the four time points (r = 0.69; *p* < 0.001). After normalisation to values obtained at the end of the vibration phase to control for changes in fibre-specific force and EMG signal characteristics, decreases in T_sust_ (−42.9%) and soleus EMG (−22.6%) remained significant and were each correlated with loss and recovery of MVC (r = 0.41 and 0.46, respectively).

**Conclusion:**

The parallel changes observed in evoked self-sustained muscle activity and force generation capacity provide motivation for future examinations on the potential influence of fatigue-induced PIC changes on motoneuron output.

## Introduction

Repeated strong muscular contractions trigger acute changes within the neuromuscular system that result in a reduced force production capacity, i.e., fatigue. During fatiguing activity, a number of supraspinal, spinal and afferent factors may contribute to, or compensate for, failure at multiple sites along the neuromuscular pathway, having an ultimate negative effect on maximal motoneuronal firing frequency and thus voluntary muscle contractile output (Taylor et al. 2016). Although the neuromuscular mechanisms contributing to the phenomenon have been studied for well over a century, little attention has been devoted to the role of persistent inward currents (PICs) in spinal motoneurons in exercise-induced fatigue. PIC activation is an important intrinsic cellular property that critically affects motoneuron firing rates (Alaburda et al. 2002; Binder 2003; Heckman et al. 2008). These persistent, depolarising currents are generated by voltage-sensitive sodium and calcium channels that can remain open as long as the membrane potential exceeds the level required for PIC activation. The amplification of synaptic input by PICs is essential for the attainment of the motor unit recruitment and firing rates necessary for high force production, with this amplification being proportional to the levels of serotonergic and noradrenergic release onto the motoneurons (Heckman et al. 2008). Given that this enhancement of synaptic input by PICs is two- to six-fold in magnitude, any loss of the contribution of PICs to motoneuron firing should substantially and negatively affect the muscular force produced for a given level of descending input (central drive) to the motoneuron pool.

Fatiguing muscular contractions can result in several acute physiological changes that might, in some cases, directly influence PIC strength. For example, a decrease in the spinal release of serotonin (Fornal et al. 2006) might lead to a withdrawal of its facilitatory effect on PICs (Goodlich et al. 2023) and thus reduce exercise capacity under some conditions. Alternatively, repeated, strong muscular contractions may release sufficient serotonin that spill-over occurs onto the axon hillock, activating 5-HT_1A_ receptors which then reduce motoneuron excitability and thus firing rates (Cotel et al. 2013; Kavanagh et al. 2019), especially during intense muscular contractions (Henderson et al. 2022). Additionally, repeated muscular contractions may reduce firing in muscle spindle endings and thus impact Ia facilitation of motoneurons (Macefield et al. 1991). Importantly, Ia activity evoked through local tendon vibration strongly activates PICs, as directly observed in animal models (Lee and Heckman 1996) and observed indirectly in humans (Gorassini et al. 1998; Trajano et al. 2014). Also, an increase in inhibitory input onto motoneurons during fatiguing exercise could decrease PIC strength as they are exquisitely sensitive to inhibition (Hounsgaard et al. 1988). Particularly, reciprocal inhibitory pathway activation has strongly and negatively affected PICs in animal (Hyngstrom et al. 2008, 2007) and human studies (Mesquita et al. 2022; Orssatto et al. 2022). Since muscular fatigue often promotes muscular co-contraction (Psek and Cafarelli 1993; Weir et al. 1998) and this antagonist muscle activation should contribute to a stronger reciprocal inhibitory drive onto agonist muscles (Crone and Nielsen 1989), PIC strength might be expected to be negatively affected.

Despite these possibilities, little is known about the effects of fatiguing muscle contractions on PIC strength in humans, despite preliminary evidence indicating a reduced PIC activity (Kirk et al. 2019; Mendes 2016). Direct PIC measurement requires invasive recordings from spinal motoneurons, and thus has so far only been achieved in animal models (Hounsgaard et al. 1988). However, distinct patterns of motor unit firing, electromyographic (EMG) activity, and torque that are likely generated by PICs have been clearly observed in humans. The paired motor unit technique (Gorassini et al. 2002a, b; Gorassini et al. 1998) is the current standard method to indirectly estimate PIC contribution to motoneuron firing in humans, with the quantification of recruitment-derecruitment hysteresis in pairs of MUs. Alternatively, neuromuscular electrical stimulation (NMES) and tendon vibration have also been used both independently (Bochkezanian et al. 2018a; Magalhães et al. 2013) and simultaneously (Bochkezanian et al. 2018b; Espeit et al. 2021; Kirk et al. 2019; Magalhães and Kohn 2010; Mesquita et al. 2021, 2022; Trajano et al. 2014) as an experimental means to generate involuntary forces that are hypothetically explicable by activation of motoneuronal PICs, such as an ongoing, or self-sustained, torque production after cessation of NMES and vibration stimuli (Mesquita et al. 2021, 2022). One issue with the assessment of self-sustained torque after simultaneous imposition of NMES and tendon vibration (i.e., the VibStim technique) after fatiguing contractions is that peripheral mechanisms of fatigue can reduce the muscle fibre force produced by a given neural stimulus (Westerblad et al. 1998). Thus, changes in self-sustained post-VibStim torques could be observed even in the absence of impaired self-sustained motoneuron firing. Moreover, a fatigue-induced reduction in muscle spindle discharge (Macefield et al. 1991) could preclude the depolarisation of Ia afferent sensory fibres during VibStim trials with a consequent decrease in the magnitude of involuntary activity, regardless of any changes in motoneuron intrinsic properties. To circumvent this, self-sustained torque and EMG activity can theoretically be normalised to the magnitude of involuntary contractions evoked by the vibration and neuromuscular stimulation itself. However, changes in these variables with fatiguing muscle contractions have yet to be assessed.

Given the above, the purposes of the present study were to determine whether: (1) fatiguing plantar flexor contractions trigger a decrease in the self-sustained torque and muscle activity measured after a bout of simultaneous plantar flexor NMES and Achilles tendon vibration, and (2) the post-fatigue recovery of voluntary muscle force is associated with a recovery of self-sustained torque and muscle activity. We tested the hypotheses that self-sustained activity would be reduced after fatiguing exercise but would recover rapidly (within minutes) after exercise cessation. Moreover, we hypothesised that positive associations would exist between changes in maximal voluntary force capacity and both self-sustained torque and EMG.

## Materials and methods

### Ethical approval

The procedures performed during this research were approved by the Edith Cowan University Human Research Ethics Committee (ID: 14127) and conformed to the standards of use of human participants in research as outlined in the *Declaration of Helsinki*, except for registration in a database. All participants were informed of the experimental procedures and gave their written consent before any testing was conducted.

### Subjects

A convenience sample of 16 adults were recruited to the study, however 3 females and 1 male did not show a sustained torque response after application of Achilles tendon with neuromuscular electrical stimulation in familiarisation (methods described in detail below; implications of the lack of response in some individuals are explored in detail in Mesquita et al. 2021, 2022, 2023). Thus, 12 men (30.7 ± 8.8 y, 1.78 ± 0.06 m, 81.2 ± 12.6 kg, 25.5 ± 2.9 kg/m^2^) completed the study in full. To be included, volunteers had to exercise less than 4 h per week and be free of medical contra-indication to physical activity. The subjects were required to abstain from taking any stimulants or depressants, including caffeine for at least 12 h and alcohol for at least 24 h prior to testing and to refrain from performing sports or hard exercise training for 24 h prior to each experimental session. All participants gave their written consent to participate in the present study.

### Experimental design

**Fig. 1:**
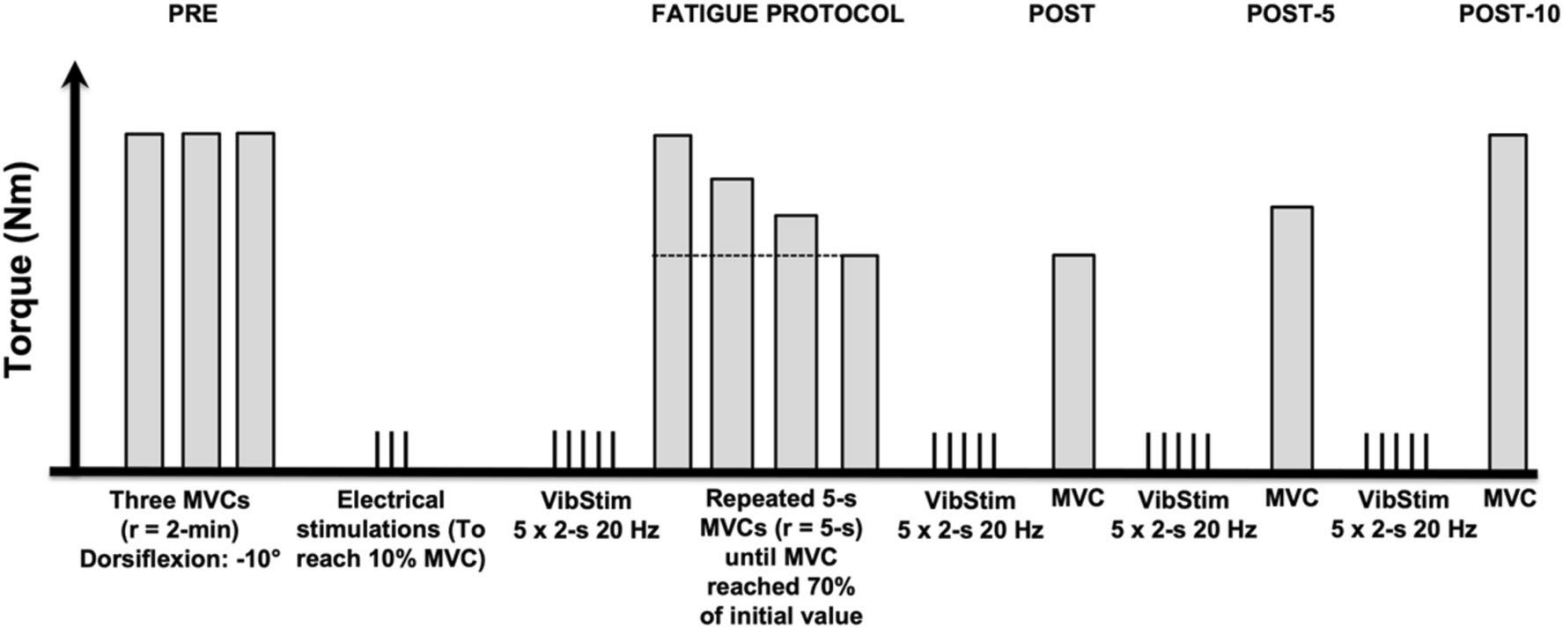
Overview of experimental protocol. Five 2-s bursts of neuromuscular electrical stimulation (20 Hz, 10% MVC) were superimposed over ongoing Achilles tendon vibration (VibStim) before (PRE) and then immediately (POST), 5-min (POST-5) and 10-min (POST-10) after fatiguing exercise consisting of 5-s plantar flexion MVCs (until maximum ankle torque decreased to 70% of maximum). *MVC* maximum voluntary contraction, *r* rest

Measurements took place on two different days at the same time of the day. The two sessions were separated by at least 48 h. The first session was used to collect anthropometric data and to familiarise the volunteers with the experimental procedures, including maximal voluntary contractions (MVC) and the electrical stimulation with tendon vibration. The second session was devoted to experimental measurements, as illustrated in Fig. 1. Before testing, the participants completed a 5-min warm-up on a stationary bicycle followed by two short bicycle sprints (~3–5 s). The participants then performed three plantar flexor isometric MVCs with a 2-min inter-trial passive rest at 10° of dorsiflexion (0° = neutral position), as described in detail below. Then, 5 × 2-s bouts of muscle electrical stimulation were delivered to the plantar flexor muscle bellies during a period of ongoing Achilles tendon vibration (VibStim) (Trajano et al. 2014), as described in detail below. The plantar flexors were chosen for study because they are primary locomotor muscles (Novacheck 1998) and have been examined previously in studies using the VibStim technique (Kirk et al. 2019; Trajano et al. 2014).

After these pre-intervention tests, the volunteers completed a fatiguing exercise protocol consisting of 5-s maximal, voluntary isometric plantar flexor contractions separated by 5 s of passive recovery until the generated torque was reduced to 70% of the initial value. MVCs were performed on an isokinetic dynamometer (Biodex System 4 Pro, Biodex, USA) with the knee fully extended (0°) and ankle in the neutral position (0°). To quantify the magnitude of fatigue and recovery, one MVC was performed immediately after completion of the exercise, and at 5 and 10 min of passive recovery. Verbal encouragement and visual feedback were provided during all voluntary MVCs. Furthermore, the VibStim technique was repeated immediately before each MVC, with this assessment being conducted immediately after the fatiguing protocol and at 5 and 10 min during recovery. EMG was recorded simultaneously from soleus (Sol), gastrocnemius medialis (GM) and tibialis anterior (TA) (described below).

### Voluntary and evoked torque measurements

Isometric voluntary and evoked torques were assessed using the isokinetic dynamometer. Participants were comfortably positioned on an adjustable chair with the hip joint flexed to 60° (0° = neutral position). The rotation axis of the dynamometer was aligned with the right lateral malleolus. Snowboard bindings were set to the footplate of the dynamometer and used to fix the foot firmly to the footplate. Before being placed in the final testing position, the seat distance from the foot plate was adjusted so the knee was flexed to ~30° with the leg relaxed, then upon straightening to place the participant in the final, testing position, the chair and footplate were flexed slightly and the mechanical compliance of the system was taken up; thus, the leg represented a strut even with the muscles relaxed (the absence of EMG activity was checked). Further flexion of the system’s components and body’s soft tissues were therefore significantly minimised (Cannavan et al. 2012). During each MVC, participants were instructed to grip the lateral seat handles to stabilise the pelvis. Torque data were digitised and exported at an analogue-to-digital conversion rate of 2 kHz (PowerLab 16/35; ADInstruments, New Zealand) using the LabChart 7.3 Pro software (ADInstruments).

### Measurement of muscle activity (EMG)

After careful preparation of the skin (shaving, light abrading with sandpaper and cleaning with alcohol) to obtain a low impedance (Z < 5 kΩ) at the skin–electrode surface, disposable bipolar electrodes (Ag–AgCl; Ambu Blue Sensor N-00-S/25, Ambu, Denmark) were positioned on Sol, GM and TA muscle bellies, according to SENIAM recommendations, with an inter-electrode distance of 10 mm. The reference electrode was attached to the lateral malleolus.

EMG signals were amplified (DualBioAmp, ML 135, ADInstruments, New Zealand) with a bandwidth frequency of 20–500 Hz (common mode rejection ratio >85 dB, gain = 1000) and simultaneously digitised together with torque signals. The sampling frequency was set at 2000 Hz. EMG activity was quantified from the root mean square (RMS) value recorded over a 500-ms analysis window during MVC where the torque was maximal (EMG_MVC_). The level of coactivation during MVC was also calculated as the ratio between TA EMG and Sol EMG (EMG_MVC,TA/Sol_) and between TA EMG and Sol + GM EMG (EMG_MVC,TA/Sol+GM_). In addition, EMG activity was measured during the VibStim protocol over a 500-ms window at the end of vibration (EMG_Vib_) and 3 s after vibration was ceased (EMG_Sust_). Sustained EMG to end-vibration EMG ratio (EMG_Sust_/EMG_Vib_) was then calculated. An overview of calculated variables and their interpretation is presented in Table 1.

**Table 1:** Overview of primary variables: abbreviations and interpretations

Variable	Abbreviation	Interpretation
Sustained torque	T_Sust_	Magnitude of sustained torque produced after cessation of the NMES + vibration; proxy for PIC activity
Sustained EMG	EMG_Sust_	Magnitude of sustained EMG after cessation of the NMES + vibration stimulus; proxy for PIC activity
Sustained-to-vibration torque ratio	T_Sust_/T_Vib_	Amplitude of sustained (persistent) torque after NMES and vibration cessation relative to torque during vibration; proxy for PIC strength, accounting for effects of ‘fatigue’
Sustained-to-vibration EMG ratio	EMG_Sust_/EMG_Vib_	Amplitude of sustained (persistent) muscle activity after NMES and vibration cessation relative to activity during vibration, indicates level of persistent motor unit firing relative to that evoked by the NMES + vibration stimulus; proxy for PIC strength, accounting for effects of ‘fatigue’
Co-activation level	EMG_MVC,TA/Sol_ or EMG_MVC,TA/Sol+GM_	Changes in the ratio relative to baseline would indicate changes in relative tibialis anterior activity; possibly affecting reciprocal inhibition onto plantar flexors

### Superimposed neuromuscular stimulation with tendon vibration (VibStim)

A combined neuromuscular electrical stimulation and tendon vibration (VibStim) technique was used to invoke involuntary plantar flexor activity (Trajano et al. 2014). This protocol has been shown to display PIC-like features such as joint angle dependence (i.e., affected by reciprocal inhibition), torque increases after repetitive activation (i.e., warm-up effect), self-sustained activity, and the inhibition of sustained firing by brief, voluntary antagonist muscle activation (Trajano et al. 2014). With the participant’s ankle set to 10° dorsiflexion, the Achilles tendon of the right leg was mechanically vibrated at 115 Hz (1-mm deflections) by a hand-held vibrator (Vibrasens, Techno Concepts, France). The vibrator was held with steady pressure against a marked point in line with the medial malleolus on the Achilles tendon for 33 s. The pressure of the vibrator alone, without mechanical vibration, did not cause any noticeable increments in resting plantar flexion torque. During the protocol, participants were instructed to hold onto the shoulder straps and not voluntarily activate the plantar- or dorsiflexor muscles. The left leg was positioned on a stool so that both legs were straight, which prevented any extraneous movement throughout the test. Real-time feedback of force and EMG data was not provided during the test, and the subjects were blinded to the outcome variables being measured.

A constant-current electrical stimulator (DS7, Digitimer, UK) was used to deliver electrical square-wave stimuli (1-ms pulse width) to the plantar flexor’s muscle belly through two self-adhesive electrodes (9 × 5 cm, Dura-Stick II, DJO Global, USA). The cathode was placed distal to the popliteal crease and the anode over the distal soleus myotendinous junction. For all electrical stimulations, the intensity necessary to reach 10% of MVC with a 0.5-s, 20-Hz tetanic stimulation was used, with this assessment done ~10 min before the VibStim protocol (Fig. 1). Ten seconds after vibration onset, five 2-s bursts of 20-Hz electrical stimulation separated by 2-s intervals were also applied. Five bursts of electrical stimulation were used because PICs tend to grow larger during repeated activation (i.e., warm-up effect) (Bennett et al. 1998; Svirskis and Hounsgaard 1997; Trajano et al. 2014), but in pilot testing this effect reached a maximum after approximately 5 stimulations.

The peak isometric plantar flexion torque, assessed during MVCs, was the only voluntary torque measurement used in this study. The involuntary “reflexive torque” was measured as the mean torque in a 500-ms window at the end of vibration (T_Vib_) and 3 s after vibration was ceased [torque sustained (T_Sust_)]. Sustained torque to end-vibration torque ratio (T_Sust_/T_Vib_) was then calculated (as summarised in Table 1). Also, the absolute difference as well as the ratio of torque during vibration after last (5th) to the first burst of electrical stimulation was calculated (500-ms window) as an indicator of a warm-up effect caused by repetitive stimulation. Visible and repeatable involuntary torque responses to vibration as well as evidence of sustained torque after vibration observed during familiarisation without the presence of fatigue were found in 12 of 16 volunteers, and these ‘responders’ subsequently formed the study cohort. The same time windows and ratios were used to compute EMG variables of Sol, GM and TA.

### Statistical analysis

Data were screened for normality of distribution and homogeneity of variances using a Shapiro–Wilk normality test and the Bartlett’s test, respectively. Separate one-way (time) analyses of variance (ANOVA) with repeated measures were used to test for changes in maximal voluntary contraction torque (MVC), EMG obtained during MVC (EMG_MVC,Sol_; EMG_MVC,GM_; EMG_MVC,TA_), the level of coactivation (EMG_MVC,TA/Sol_; EMG_MVC,TA/Sol+GM_), the magnitude of relative and absolute warm-up effect, sustained torque (T_Sust_), sustained Sol EMG (EMG_Sust,Sol_), sustained GM EMG (EMG_Sust,GM_), sustained TA EMG (EMG_Sust,TA_), sustained torque to end-vibration torque ratio (T_Sust_/T_Vib_), and sustained EMG to end-vibration EMG ratio for Sol (EMG_Sust,Sol_/EMG_Vib,Sol_), GM (EMG_Sust,GM_/EMG_Vib,GM_) and TA (EMG_Sust,TA_/EMG_Vib,TA_) using Statistica 12.0 software (Statsoft, Inc, USA). When the ANOVA revealed a significant effect, a Fisher’s LSD post hoc test was used for post hoc comparisons, with effect sizes reported as eta squared (η^2^). The limit for statistical significance was set at *p* < 0.05.

Correlations for repeated observations (within-subjects) were calculated to examine relationships between changes in MVC torque and changes in T_sust_, EMG_Sust,Sol_, EMG_Sust,GM_, T_sust_/T_Vib_, EMG_Sust,Sol_/EMG_Vib,Sol_ and EMG_Sust,GM_/EMG_Vib,GM_ over the fatigue and recovery periods (i.e. across the four time points) using the method described by Bland and Altman (1995). For this, variables calculated at all four time points were input into a univariate ANOVA with either MVC as the dependent variable, ‘participant’ as the fixed factor, and the dependent variables of T_sust_, EMG_Sust,Sol_, EMG_Sust,GM_, T_sust_/T_Vib_, EMG_Sust,Sol_/EMG_Vib,Sol_ and EMG_Sust,GM_/EMG_Vib,GM_ as covariates using Statistica 12.0 software (Statsoft, Inc, USA). The correlation coefficient was then calculated from the ANOVA output table using the Type III errors in the equation: $\sqrt{\frac{Sumof squares (independent variable)}{Sum of squares \left(independent\right)+Sum of squares TotalError}}$

The p-value was obtained from the analysis of variance table for the *F* test.

## Results

### Fatigue protocol outcomes

18.6 ± 7.1 (range: 11–33) repetitions were performed in the fatigue protocol (i.e. to 70% MVC).

### Changes in maximum voluntary torque (MVC) and EMG amplitude

**Fig. 2:**
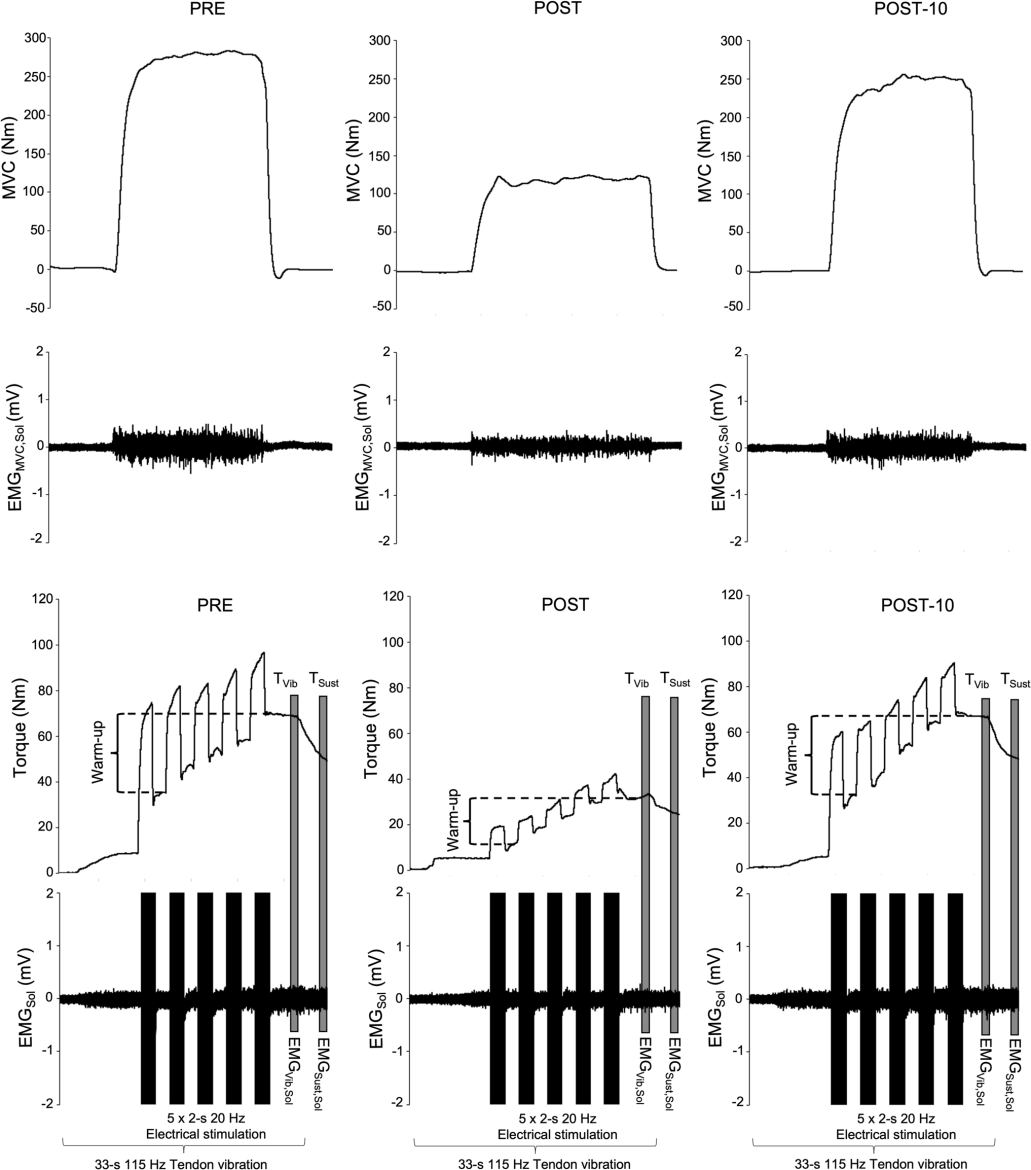
Example raw data for one individual before (PRE) and both immediately (POST) and 10 min after (POST-10) completion of the fatiguing, maximal plantar flexion contractions. The loss (to POST) and recovery (to POST-10) of maximal voluntary contraction torque (MVC) and soleus electromyogram amplitudes (EMG) measured during MVC (EMG_MVC,Sol_) were associated with reductions in, and recovery of, torque and EMG_Sust,Sol_ during the period of sustained torque measured after cessation of VibStim. *T* torque, *Sust* value (torque or EMG) representing sustained activity obtained 3-s after the end of vibration, *Vib* Value obtained at the end of vibration period

An example of raw MVC, T_Vib_, T_sust_ and EMG data obtained before (PRE), and immediately (POST), and 10 min (POST-10) after the fatiguing exercise is shown in Fig. 2. There was a significant time effect for MVC (F(3,33) = 15.6, *p* < 0.001, η^2^ = 0.58) with a decrease from PRE to POST (∆ = −25.6 ± 17.9%, *p* < 0.001) and POST-5 (∆ = −9.1 ± 10.3%, *p* = 0.027) but recovery by POST-10 (∆ = −6.4 ± 6.7%, *p* = 0.126), as shown in Table 2. These changes occurred alongside changes in EMG_MVC,Sol_ (F(3,33) = 11.1, *p* < 0.001, η^2^ = 0.50), including a significant decrease from PRE to POST (∆ = −23.4 ± 21.0%, *p* < 0.001) and POST-5 (∆ = −11.6 ± 13.1%, *p* = 0.0017) but recovery by POST-10 (∆ = −0.9 ± 12.5%, *p* = 0.46). There was also a significant time effect for EMG_MVC,GM_ (F(3,33) = 7.3, *p* < 0.001, η^2^ = 0.40), with a decrease at POST (∆ = −30.0 ± 20.7%, *p* < 0.001) but recovery by POST-5 (∆ = −13.7 ± 11.2%, *p* = 0.09) and POST-10 (∆ = −3.8 ± 17.6%, *p* = 0.66). Finally, a significant time effect was observed for EMG_MVC,TA_ (F(3,33) = 7.3, *p* < 0.001, η^2^ = 0.40), with a significant decrease from PRE to POST (∆ = −29.6 ± 34.0%, *p* < 0.001) and POST-5 (∆ = −11.2 ± 23.8%, *p* = 0.048) but recovery by POST-10 (∆ = −5.5 ± 22.1%, *p* = 0.28). No significant time effect was observed for the ratios EMG_MVC,TA/Sol_ (F(3,33) = 2.4, *p* = 0.087, η^2^ = 0.18) or EMG _MVC,TA/Sol+GM_ (F(3,33) = 0.74, *p* = 0.53, η^2^ = 0.06).

**Table 2:** Torque and electromyogram (EMG) data obtained before and after fatiguing voluntary plantar flexor contractions using maximal voluntary contractions (MVC) and superimposed muscle stimulation with tendon vibration (VibStim)

Variable	PRE	POST	POST-5	POST-10
	*Voluntary measurements*			
MVC (Nm)	239.2 ± 59.5	174.8 ± 51.0***	216.4 ± 55.5*	223.7 ± 58.8
EMG_MVC,Sol_ (mV)	0.197 ± 0.082	0.145 ± 0.055***	0.173 ± 0.069*	0.190 ± 0.068
EMG_MVC,GM_ (mV)	0.319 ± 0.145	0.218 ± 0.120***	0.278 ± 0.140	0.309 ± 0.150
EMG_MVC,TA_ (mV)	0.037 ± 0.018	0.022 ± 0.014***	0.030 ± 0.014*	0.033 ± 0.016
EMG_MVC,TA/Sol_	0.222 ± 0.134	0.180 ± 0.112	0.209 ± 0.124	0.200 ± 0.116
EMG_MVC,TA/Sol+GM_	0.078 ± 0.041	0.070 ± 0.046	0.075 ± 0.040	0.072 ± 0.036
	*Evoked measurements*			
T_sust_ (Nm)	22.6 ± 22.0	9.9 ± 12.2***	19.1 ± 17.7	23.8 ± 22.5
T_Sust_ /T_Vib_	0.594 ± 0.222	0.352 ± 0.268***	0.501 ± 0.266	0.594 ± 0.240
EMG_Sust,Sol_ (mV)	0.032 ± 0.012	0.018 ± 0.009***	0.027 ± 0.014	0.032 ± 0.012
EMG_Sust,GM_ (mV)	0.025 ± 0.024	0.015 ± 0.017	0.021 ± 0.016	0.024 ± 0.023
EMG_Sust,TA_ (mV)	0.006 ± 0.003	0.006 ± 0.004	0.006 ± 0.002	0.006 ± 0.002
T_WarmAbs_ (Nm)	12.7 ± 5.7	8.7 ± 10.3	14.2 ± 9.9	16.6 ± 12.4
EMG_WarmAbs,Sol_ (mV)	0.020 ± 0.014	0.011 ± 0.007***	0.016 ± 0.010	0.021 ± 0.018
EMG_WarmdAbs,GM_ (mV)	0.009 ± 0.009	0.000 ± 0.009*	0.010 ± 0.016	0.013 ± 0.019
EMG_WarmAbs,TA_ (mV)	0.029 ± 0.097	−0.004 ± 0.015	0.019 ± 0.064	0.019 ± 0.064
T_WarmRatio_	4.1 ± 3.9	6.0 ± 10.7	1.5 ± 4.0	3.2 ± 2.9
EMG_WarmRatio,Sol_	1.9 ± 0.9	1.7 ± 0.8	1.9 ± 0.9	1.8 ± 0.6
EMG_WarmRatio,GM_	1.6 ± 0.8	1.5 ± 1.1	1.7 ± 0.7	1.6 ± 0.9
EMG_WarmRatio,TA_	1.2 ± 0.4	1.0 ± 0.1	1.2 ± 0.3	1.1 ± 0.3
EMG_Sust,Sol_/EMG_Vib,Sol_	0.716 ± 0.186	0.552 ± 0.211***	0.642 ± 0.197	0.697 ± 0.194
EMG_Sust,GM_/EMG_Vib,GM_	0.707 ± 0.327	0.544 ± 0.330	0.575 ± 0.268	0.634 ± 0.271
EMG_Sust,TA_/EMG_Vib,TA_	0.892 ± 0.278	0.968 ± 0.082	0.886 ± 0.281	0.868 ± 0.274

Mean ± SD

*PRE* Before fatigue, *POST* immediately after fatigue, *POST-5* 5-min after fatigue, *POST-10* 10-min after fatigue, *Sol* soleus, *GM* gastrocnemius medialis, *TA* tibialis anterior, *MVC* maximal voluntary contraction, *T* torque, *Sust* value (torque or EMG) representing sustained activity obtained 3-s after the end of vibration, *Vib* Value obtained at the end of vibration period, *Warm* Warm-up effect

Significantly different from PRE at *p < 0.05; ***p < 0.001, respectively

### Changes in self-sustained and warm-up torque and EMG

**Fig. 3:**
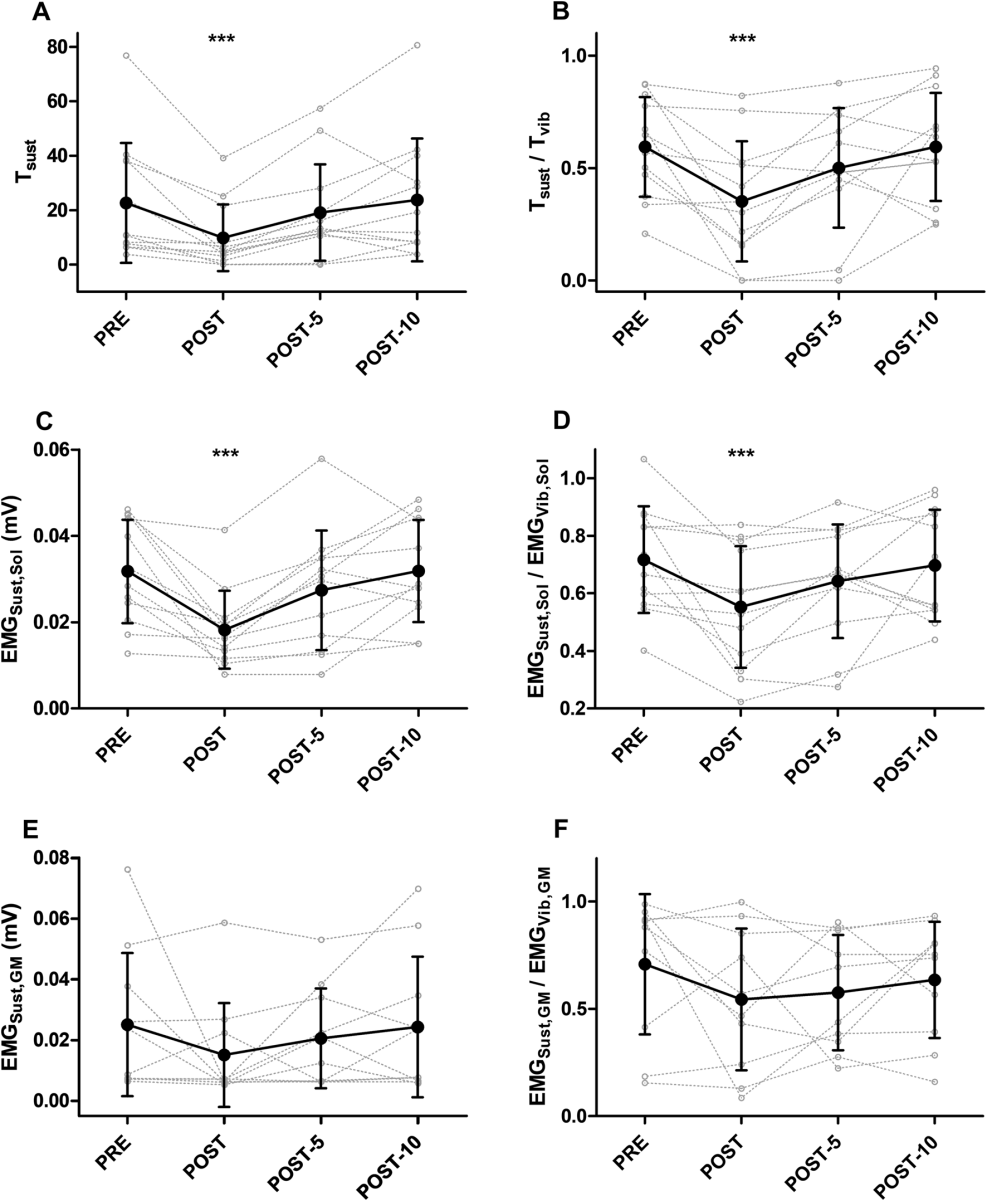
Changes in sustained torque (T_Sust_; Panel **A**), sustained torque to end-vibration torque ratio (T_Sust_/T_Vib_; Panel **B**), soleus sustained EMG (EMG_Sust,Sol_; Panel **C**), soleus sustained EMG to end-vibration EMG ratio (EMG_Sust,Sol_/EMG_Vib,Sol_; Panel **D**), gastrocnemius medialis sustained EMG (EMG_Sust,GM_; Panel **E**), and gastrocnemius medialis (GM) sustained EMG to end-vibration EMG ratio (EMG_Sust,GM_/EMG_Vib,GM_; Panel **F**) recorded during the period of self-sustained muscle activity. The loss and recovery of maximal voluntary contraction (MVC) torque was temporally associated with a loss and recovery of both the torque and soleus EMG recorded during the period of sustained torque production, as evidenced by changes in T_Sust_, T_Sust_/T_Vib_, EMG_Sust,Sol_, and EMG_Sust,Sol_/EMG_Vib,Sol_. ***Significantly different from PRE at *p* < 0.001

A significant time effect was observed for sustained torque (T_sust_; F(3,33) = 8.2, *p* < 0.001, η^2^ = 0.43), with significant decreases from PRE to POST (∆ = −59.0 ± 31.1%, *p* < 0.001) but recovery by POST-5 (∆ = −0.1 ± 68.8%, *p* = 0.26) and POST-10 (∆ = +17.4 ± 76.2%, *p* = 0.72) (Table 2; Fig. 3). EMG_Sust,Sol_ changed significantly over time (F(3;33) = 11.1, *p* < 0.001, η^2^ = 0.50), decreasing from PRE to POST (∆ = −38.4 ± 24.8%, *p* < 0.001) but recovering by POST-5 (∆ = −10.3 ± 33.2%, *p* = 0.12) and POST-10 (∆ = 3.6 ± 25.1%, *p* = 0.97) (Fig. 3). No significant time effects were detected for EMG_Sust,GM_ (F(3;27) = 1.3, *p* = 0.28, η^2^ = 0.13) or EMG_Sust,TA_ (F(3,33) = 0.14, *p* = 0.93, η^2^ = 0.01).

The absolute warm-up torques (T_Warm,Abs_), calculated as the absolute difference of torque obtained during the vibratory period after the 5th (final) versus the 1st stimulation, did not statistically change over time (F(3;33) = 2.39, *p* = 0.087, η^2^ = 0.18), with differences from PRE to POST of −24.4 ± 75.6%, to POST-5 of +29.1 ± 81.9% and to POST-10 of +64.5 ± 155.5%. Nonetheless, statistical changes were observed in EMG_Warm,Abs,Sol_ (F(3,33) = 3.1, *p* = 0.039, η^2^ = 0.22), with a decrease from PRE to POST (∆ = −35.8 ± 46.8%, *p* < 0.001) and recovery by POST-5 (∆ = −10.3 ± 49.0%, *p* = 0.29) and POST-10 (∆ =  +8.8 ± 67.3%, *p* = 0.76) (Table 2). The same result was obtained for EMG_Warm,Abs,GM_ (F(3,33) = 4.2, *p* = 0.012, η^2^ = 0.28), with a significant decrease from PRE to POST (∆ = −50.5 ± 162.4%, *p* = 0.029) and recovery by POST-5 (∆ =  +229.8 ± 450.1%, *p* = 0.69) and POST-10 (∆ =  +109.0 ± 395.1%, *p* = 0.30). No significant time effect was observed for EMG_Warm,Abs,TA_ (F(3,33) = 1.04, *p* = 0.39, η^2^ = 0.09).

When the warm-up was calculated as a ratio of the torque or EMG data obtained during the vibratory period after the 5th (final) versus the 1st stimulation, no significant time effect was observed for torque (T_Warm,Ratio_; F(3,33) = 1.4, *p* = 0.27, η^2^ = 0.11) or EMG_Warm,Ratio,Sol_ (F(3,33) = 0.50, *p* = 0.69, η^2^ = 0.04), EMG_Warm,Ratio,GM_ (F(3,33) = 0.26, *p* = 0.86, η^2^ = 0.02), and EMG_Warm,Ratio,TA_ (F(3,33) = 1.8, *p* = 0.17, η^2^ = 0.14).

### Changes in the ratios of self-sustained torque and EMG to end-vibration torque and EMG

A significant time effect was observed for T_Sust_/T_Vib_ (F(3,33) = 7.1, *p* < 0.001, η^2^ = 0.39), with a significant decrease from PRE to POST (∆ = −42.9 ± 37.8%, *p* < 0.001) but recovery by POST-5 (∆ = −14.4 ± 46.8%, *p* = 0.13) and POST-10 (∆ =  +1.6 ± 23.8%, *p* = 0.99) (Table 2; Fig. 3). These changes occurred alongside changes in EMG_Sust,Sol_/EMG_Vib,Sol_ (F(3,33) = 5.3, p = 0.0044, η^2^ = 0.32), including a significant decrease from PRE to POST (∆ = −22.6 ± 22.9%, *p* < 0.001) and recovery by POST-5 (∆ = −8.9 ± 23.5%, *p* = 0.11) and POST-10 (∆ = −2.6 ± 10.8%, *p* = 0.66) (Fig. 3). In contrast, no statistical changes were observed in EMG_Sust,GM_/EMG_Vib,GM_ (F(3,27) = 1.47, *p* = 0.24, η^2^ = 0.14) or EMG_Sust,TA_/EMG_Vib,TA_ (F(3,33) = 0.99, *p* = 0.41, η^2^ = 0.08).

### Temporal relationships between changes in MVC and changes in sustained torque and EMG variables

A significant within-subjects correlation was observed between changes in MVC and changes in T_Sust_ when computed across the four time points (i.e., to fatigue and through recovery; r = 0.69; *p* < 0.001). A significant within-subjects correlation was also observed between changes in MVC and changes in EMG_Sust,Sol_ (r = 0.62; *p* < 0.001). However, no correlation was observed between changes in MVC and changes in EMG_Sust,GM_ (r = 0.14, *p* = 0.15).

**Fig. 4:**
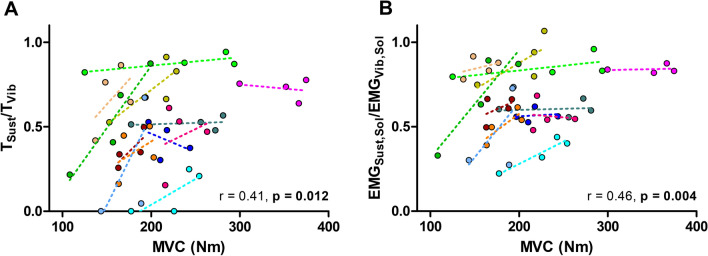
Relationships between changes in maximum voluntary contraction torque (MVC) and (**A**) the ratio of the torques produced during the sustained torque period to the vibration period, and (**B**) the ratio of the soleus EMG produced during the sustained torque period to the vibration period (within-subjects analyses). (**A**) Changes in T_Sust/TVib_ over time were correlated with temporal changes in MVC, and (**B**) changes in EMG_Sust,Sol_/EMG_Vib,Sol_ over time were correlated with temporal changes in MVC. T_Sust_, sustained torque; T_Vib_, torque during vibration after NMES cessation; EMG_Sust,Sol_, soleus EMG amplitude during the sustained torque period; EMG_Vib,Sol_, soleus EMG amplitude during vibration after NMES cessation

As shown in Fig. 4A, a significant within-subjects correlation was also observed between changes in MVC and changes in T_sust_/T_Vib_ (r = 0.41, *p* = 0.012) when computed across the four time points. Also, for EMG variables, a significant correlation was observed between changes in MVC and changes in EMG_Sust,Sol_/EMG_Vib,Sol_ (r = 0.46, *p* = 0.004; Fig. 4B). However, no correlation was observed between changes in MVC and changes in EMG_Sust,GM_/EMG_Vib,GM_ (r = 0.13, *p* = 0.15).

A significant within-subjects correlation between the change in Sol EMG during MVC and its change during the sustained torque period of the VibStim test was also observed regardless of whether absolute (r = 0.70, *p* < 0.001) or normalised (r = 0.67, *p* < 0.001) values were used. Similarly, significant within-subjects correlations were observed for GM EMG when using absolute (r = 0.19, *p* = 0.025) and normalised (GM: r = 0.435, *p* = 0.014) values.

## Discussion

Highly fatiguing, maximal-effort, plantar flexion contractions reduced maximal ankle torque production (to <70% MVC) as well as both the torque (−59.0%) and soleus muscle activity (−38.4%) recorded during the sustained contraction that continued after the tendon vibration-NMES stimulus (VibStim) was removed. This fatigue-induced reduction in self-sustained muscle activity may reflect a reduced PIC activity in spinal motoneurons (Mesquita et al. 2021, 2022; Trajano et al. 2014) as it was still observed even after the potential effect of other neuromuscular confounding factors was accounted for. Moreover, the temporal association between the decrease and recovery of MVC torque and changes in sustained torque (absolute: r = 0.69; normalised: r = 0.41) and soleus EMG (absolute: r = 0.62; normalised: r = 0.46) is also provocative and should motivate further studies to examine whether the reduced ability to generate voluntary force after fatiguing exercise partly results from a decrease in the contribution of PICs to motoneuron firing.

It should be noted that other mechanisms unrelated to motoneuronal PICs may speculatively contribute to a reduction in VibStim variables after a fatiguing protocol. First, peripheral fatigue alters muscle fibre force and thus the absolute magnitude of the involuntary torque (Westerblad et al. 1998), and possibly the magnitude of muscle potentiation mechanisms (Blazevich and Babault 2019; Frigon et al. 2011). Second, a decrease in muscle spindle discharge due to fatigue (Macefield et al. 1991) might hinder the depolarisation of Ia afferent sensory fibres during VibStim, leading to a decline in the extent of involuntary torque and EMG. Third, fatigue-induced decreases in the velocity of propagation of the action potentials in the muscle fibres (Farina et al. 2006), and preferential changes in the shapes of action potentials of higher-threshold motor units (Enoka et al. 1992) could increase the chance of EMG amplitude cancellation (Keenan et al. 2005) independent of changes in the magnitude of neural drive to the muscle. In an attempt to circumvent these issues, both the sustained torque and EMG activity were normalised to the signal amplitudes measured at the end of the vibration phase, since any loss in fibre-specific force or unrelated changes in EMG characteristics should be accounted for. This secondary analysis still revealed a significant decrease and then recovery of sustained torque (−42.9%) and soleus EMG (−22.6%) across time points, which were correlated with the loss and recovery of MVC torque (r = 0.41 and 0.46, respectively); gastrocnemius medialis EMG was not statistically reduced during the sustained torque period. Additionally, post-hoc analysis revealed a correlation between the change in soleus EMG during MVC and its change during the sustained torque period of the VibStim test, regardless of whether absolute (r = 0.70, *p* < 0.001) or normalised (r = 0.67, *p* < 0.001) VibStim EMG values were assessed. Given that the magnitude of involuntary activity at the end of the vibration is lower after the fatiguing protocol, one could have hypothesised that floor effects would not allow us to observe reductions in the computed VibStim ratios. Floor effects could be important confounding factors in the interpretation of VibStim variables, as previously discussed in relation to other interventions (Mesquita et al. 2022). Nonetheless, significant reductions in these ratios were observed, and it may be postulated that these ratios are sufficiently sensitive to changes in the contribution of PICs to self-sustained firing. The present results are also consistent with previous, preliminary findings in which estimated PICs at the soleus motoneurons were impaired by fatiguing, maximal-effort contractions, which then impacted maximal force production capacity (Kirk et al. 2019; Mendes 2016).

In fact the present data and those presented by others indicate that soleus motor units may be particularly susceptible to intervention, given that (1) decreased soleus but not gastrocnemius medialis self-sustained EMG (Trajano et al. 2014) and delta F (firing rate hysteresis; Trajano et al. 2020) are observed after brief (5-min) muscle stretching, that (2) increases in contraction force robustly increase delta F in soleus but has less effect in gastrocnemius medialis (Orssatto et al. 2021), and that (3) jaw clenching and mental stress also strongly enhance self-sustained soleus EMG (although effects on GM were not assessed; Mesquita et al. 2023). The reasons for such susceptibility are yet to be fully elucidated, although the limited common drive observed between triceps surae components (Hug et al. 2021) may allow for muscle-specific variation in PIC amplitudes to arise, and potentially for differential influences of inhibitory-facilitatory drive onto the muscles to play a role.

In the present study tendon vibration was used to excite the motoneurons, as previously done in animal models (Lee and Heckman 1996), and then neuromuscular electrical stimulation was imposed to trigger additional reflexive recruitment of motoneurons via direct depolarisation of Ia afferents. Hallmarks of PIC formation were then clearly observed, including a progressive increase in torque during the trials consistent with calcium-dependent warm-up observed during repetitive activations (Svirskis and Hounsgaard 1997) and both persisting torque and muscle activity after stimulation cessation, which could be caused by PIC-related bistable behaviour (Lee and Heckman 1998) (see Table 2). In previous studies, other hallmarks of PICs have been readily observed when using the VibStim protocol, including joint angle (i.e., antagonist muscle length) dependence (Hyngstrom et al. 2007; Trajano et al. 2014), and the termination of sustained firing by reciprocal inhibition (Hounsgaard et al. 1988; Mesquita et al. 2022). As the protocol does not require voluntary muscle contraction, outcomes should not be influenced by alterations in voluntary muscle activation-dependent monoamine release (Noga et al. 2017; Veasey et al. 1995). Moreover, VibStim variables appear to be sensitive to interventions that would be expected to alter PIC strength. For example, ongoing (self-sustained) ankle plantar flexor torque and EMG were reduced after 5 min of plantar flexor muscle stretching and then recovered in the 15-min post-stretch period (Trajano et al. 2014), with similar changes also being reported when PICs were estimated by the paired motor unit technique (Trajano et al. 2020), and possibly attributed to reductions in noradrenergic amplification of PICs (Trajano and Blazevich 2021). Furthermore, whole-body relaxation might decrease neuromodulatory input (Teixeira et al. 2005) and it was recently shown to decrease self-sustained plantar flexor torque and EMG activity in individuals who had strong pre-intervention responses to VibStim (Mesquita et al. 2022). At least in some cases, VibStim may then provide an indirect estimation of PIC activity in the absence of voluntary drive to the muscle using equipment readily available in many laboratories and clinical environments.

It may be postulated that decreases in the VibStim ratios computed in this study are more indicative of changes in the contribution of PICs to self-sustained firing in lower, rather than higher-threshold motor units. Longer durations of self-sustained firing after cessation of excitatory synaptic input have been found in low-threshold than higher-threshold motor units (Lee and Heckman 1998). Moreover, a lower proportion of higher-threshold motor units could be expected to be active after fatigue (Grimby et al. 1981). This could potentially be confirmed in future studies by decomposing EMG signals during VibStim to identify motor unit firing and estimate recruitment thresholds via subsequent ramp contractions. Subsequently, further optimisation of VibStim methods (Mesquita et al. 2021) may allow the examination of a greater proportion of the motoneuron pool, including the motoneurons that do not exhibit pronounced bistable behaviour (Lee and Heckman 1998). This might improve our understanding of the modulation of motoneuronal self-sustained firing in fatigue and other contexts.

The potential reduction in the PIC contribution to motoneuron firing in this study might have occurred for several reasons, including reduced serotonin release (Fornal et al. 2006; Goodlich et al. 2023), serotonin spill over from the somato-dendritic compartment onto inhibitory 5-HT_1a_ receptors at the axon initial segment of the motoneurons (Cotel et al. 2013; Henderson et al. 2022; Kavanagh et al. 2019), greater levels of co-contraction induced reciprocal inhibition (Crone and Nielsen 1989; Mesquita et al. 2022), and/or the inhibitory actions of fatigue-sensitive group III/IV afferents (Martin et al. 2006). Nonetheless, it has to be considered that the reductions in self-sustained PIC-like patterns could theoretically have been caused by alterations that would be independent of motoneuron intrinsic excitability. A study conducted on adult cats (Mendez-Fernandez et al. 2019) suggests that afferent stimulation may cause self-sustained firing in spinal cord interneurons, in addition to, or instead of, alpha motoneurons, which could be a result of reverberating spinal circuits (Bellardita et al. 2017). Changes in the resting firing rates of muscle spindles might also be speculated to explain the observed changes in self-sustained activity, although this phenomenon (intrafusal stiction) is typically seen after voluntary isometric contraction (Gregory et al. 1990; Wilson et al. 1995) and would not be expected to occur in the reflexive contractions observed in VibStim. In fact, muscle spindles are known to show a reduced spontaneous firing rate immediately after the cessation of vibration (Ribot-Ciscar et al. 1998).

## Conclusions

The present results demonstrate that self-sustained firing of MUs, a hallmark of PIC activation, is reduced after a series of maximal, fatiguing muscle contractions is performed. The findings are of both practical and scientific importance since they provide evidence that a rarely examined mechanism may contribute to muscle functional loss and recovery after intense, fatiguing muscle contractions. Because (especially Ca^2+^-dependent) PIC activation is a relatively slow process (e.g., PIC-induced initial acceleration of motoneuron firing lasts ~1 to 2 s) (Binder et al. 2020), estimates of changes in PIC strength are not likely to be observed in tests in which brief action potential volleys are induced in the corticospinal pathway, such as during stimulations that elicit motor evoked potentials (transcranial, cervicomedullary or thoracolumbar stimulation) or H-reflexes. Thus, important acute physiological adaptations at the motoneuron in response to repeated activations, such as changes in PIC strength, will not have been detected in studies using these tests (see Kalmar 2018 for discussion). From a practical perspective it is of both scientific and practical benefit to describe fatigue-induced neuromuscular changes and the underlying causes of reductions in PIC activity in order to motivate the development of interventions that mitigate their loss in exercise in health and in the many disorders for which fatigue is a major symptom. Previous evidence, for example, indicates that stimulants (Udina et al. 2010; Walton et al. 2002) and remote voluntary contractions (Mackay Phillips et al. 2023; Orssatto et al. 2022) present opportunities. Nonetheless, since the present data provide only indirect evidence of changes in PIC behaviour, it will be of interest to further study these effects as methods become more advanced (Beauchamp et al. 2023), and in combination with the examination of a portion of motoneurons actively contributing to voluntary contractions via the well-established paired motor unit technique (Afsharipour et al. 2020).

## Data Availability

The datasets generated during and/or analysed during the current study are available in the GitHub repository, https://github.com/ricardoNOmesqui/FatigueVibStim.git.

## Abbreviations

Abs: Absolute (non-normalised) value of a variable
EMG: Electromyogram (amplitude)
GM: Gastrocnemius medialis
MU: Motor unit
MVC: Maximal voluntary contraction
NMES: Neuromuscular electrical stimulation
PIC: Persistent inward current
PRE: Before fatiguing contractions
POST (-5, -10): After fatiguing contractions (5 and 10 min)
Ratio: Value after 5th divided by value after 1st muscle stimulation
Sol: Soleus
Sust: Sustained (period after NMES and vibration cessation)
TA: Tibialis anterior
Vib: Vibration (period after NMES with ongoing vibration)
VibStim: Method using tendon vibration superimposed by stimulation (NMES)
Warm: Warm-up (increase in torque output with repeated NMES stimulations)
